# Temperature Sensitivity Characteristics of SBS/CRP-Modified Bitumen after Different Aging Processes

**DOI:** 10.3390/ma11112136

**Published:** 2018-10-30

**Authors:** Rui He, Shuhua Wu, Xiaofeng Wang, Zhenjun Wang, Huaxin Chen

**Affiliations:** 1 School of Materials Science and Engineering, Chang’an University, Xi’an 710061, Shaanxi, China; heruia@163.com (R.H.); hxchen@chd.edu.cn (H.C.); 2 Engineering Research Center of Transportation Materials, Ministry of Education, Chang’an University, Xi’an 710064, Shaanxi, China; 3 School of Civil and Architectural Engineering, Zhengzhou University of Aeronautics, Zhengzhou 450046, Henan, China; wshuhua0506@126.com; 4 Henan Provincial Communications Planning & Design Institute Co., Ltd., Zhengzhou 450052, Henan, China

**Keywords:** crumb rubber powder, SBS/CRP-modified bitumen, aging processes, temperature sensitivity characteristics

## Abstract

Temperature sensitivity characteristics of bitumen can be evidently influenced by modifier types and natural aging processes. Many types of modifiers have been used to improve the temperature sensitivity performance of bitumen, but their effects are different. Therefore, different bitumen specimens as well as SBS/CRP (Styrene-butadiene-styrene polymer/crumb rubber powder)-modified bitumen were prepared and the temperature sensitivity characteristics of bitumen after different aging processes were analyzed in this study. A dynamic rheological property test and performance test at low temperature were carried out to analyze temperature sensitivity and low temperature rheological properties of bitumen. An infrared spectrum test was adopted to study the effect of functional groups under different aging process on the properties of bitumen. The relationship between macroscopic properties and microstructures of bitumen was analyzed. The results show that SBS/CRP-modified bitumen has a strong anti-aging ability in that its flexibility and structure remain in a good condition after long-term aging. The aging process has no significant effect on SBS/CRP-modified bitumen. SBS/CRP-modified bitumen has an excellent low-temperature relaxation ability and low-temperature crack resistance. In contrast to original bitumen and SBS-modified bitumen, the temperature sensitivity performance of SBS/CRP-modified bitumen is evidently enhanced. The physical blending effect is dominant in the bitumen modified process and there is no evident chemical reaction between bitumen and crumb rubber powder. SBS/CRP-modified bitumen is recommended for wide use in plateau areas with ultraviolet and cold surroundings.

## 1. Introduction

Styrene-butadiene-styrene polymer (SBS)-modified bitumen is widely used in highway construction due to its excellent high-temperature stability, low-temperature cracking resistance, aging resistance, and fatigue resistance [1,2,3]. However, due to the existence of the SBS carbon-carbon double bond, its low-temperature performance and aging resistance are slightly inadequate when it is used in special areas such as plateau areas with cold and ultraviolet surroundings [4,5]. Rasool, et al. [6] analyzed the aging and rheological properties of waste rubber modified bitumen. The results showed that rubber powder could further enhance the ductility and anti-aging properties of the SBS-modified mechanism. Liang, et al. [7] studied the thermal rheology and compatibility of SBS-modified bitumen with different S and B segment ratios. The results showed that SBS-modified bitumen with a 30% S segment had excellent viscoelasticity and low-temperature sensitivity; the compatibility of SBS-modified bitumen became worse with the increase of the S segment ratio.

However, the demand for SBS modifier is increasing and leads to an increase in price with the development of highway construction. At the same time, SBS polymer is difficult to recycle, which is not conducive to sustainable development. Therefore, the use of rubber powder instead of SBS modifier has become popular. Some studies have shown that crumb rubber powder (CRP) and original bitumen can produce crack passivation at their interface and consume energy. Moreover, flexibility, elastic recovery, anti-aging, oxidation resistance, and many other advantages have been improved for original bitumen [8,9,10,11,12]. Crumb rubber powder modified bitumen can improve the high-temperature performance, rutting resistance, and aging resistance of bitumen pavement and can reuse waste rubber [13,14,15]. However, the requirement for rubberized bitumen pavement is becoming higher and higher with the development of bitumen pavement construction. Moreover, the low-temperature performance of rubber modified bitumen can not meet the requirements for bitumen pavement. On the other hand, the aging resistance of SBS-modified bitumen is not as good as that of rubber modified bitumen. Therefore, SBS/CRP-modified bitumen can not only reduce the cost of SBS modifier, but can also improve the modified effect and promote the recycling of resources.

Rubber powder is the main factor affecting the anti-aging performance of SBS/CRP-modified bitumen; and the content of rubber powder is related to the temperature sensitivity of modified bitumen. Researchers tend to focus on single performance studies and ignore the impact of performance. Therefore, it is still necessary to explore the temperature characteristics of SBS/rubber powder SBS/CRP-modified bitumen under different aging conditions. Guo, et al. [16,17] studied the best preparation technology of SBS/rubber powder SBS/CRP-modified bitumen. Li, et al. [18,19] found that SBS/CRP-modified bitumen could improve high-temperature stability. Li, et al. [20] studied the aging resistance of SBS/rubber powder SBS/CRP-modified bitumen and found that the aging resistance of SBS/rubber powder SBS/CRP-modified bitumen was inferior to that of rubber powder modified bitumen. Tan, et al. [21] found that rubber powder (or SBS) and aromatic oil could significantly improve the elastic energy storage of bitumen. Wang, et al. [22] prepared the SBS/CRP-modified bitumen with SBS and rubber powder and obtained the best preparation process. In order to improve the aging resistance of bitumen, inorganic or organic powders such as rubber powder, nano-TiO_2_ and carbon black can be added. Rossi, et al. [23,24] studied natural resources such as phospholipids, ascorbic acid and organosilane, polyphosphoric acid, food grade phospholipids were homogeneously mixed to bitumen to improve aging resistance. However, these modified bitumens have a high viscosity, so it is difficult to use them in cold areas [25]. However, single modified bitumen can not meet the requirements of bitumen binder in special areas where temperature changes sharply.

The objectives of this study are to improve the temperature sensitivity and anti-aging ability of bitumen. Therefore, SBS and CRP were used to modify the bitumen taking into account material performance in this study. Softener was added to reduce the viscosity of the SBS/CRP-modified bitumen, and a variety of additives were added to improve the stability and aging resistance of the modified bitumen, which was prepared to improve the low-temperature performance and anti-aging performance of the modified bitumen. The physical properties and low-temperature rheological properties of SBS/RP-modified bitumen before and after the thin film oven test (TFOT) and pressurized aging vessel (PAV) aging were analyzed. Based on the infrared spectroscopy, the relationship between macroscopic properties and microstructure was explored; then, the anti-aging and low-temperature properties of SBS/RP-modified bitumen were evaluated.

## 2. Experimental Stage

### 2.1. Raw Materials

The properties of original bitumen are shown in Table 1. SBS was used as a modifier and the amount of SBS was 4.5% in bitumen weight. Rubber powder with a 40–60 mesh was used and its main properties are given in Table 2. Solvent enhancers were aromatic oils rich in saturated and aromatic components, which can make rubber powder and SBS swell sufficiently in bitumen and reduce the viscosity of bitumen. Its density was 1.10 g/cm^3^ and the content was 7% in bitumen weight, respectively. The density of the anti-aging agent was 0.94 g/cm^3^ and its content was 3% in bitumen weight. The stabilizer was sulfur powder and the amount was 0.2% in bitumen weight.

### 2.2. Preparation of SBS/CRP-Modified Bitumen

**Figure 1:**
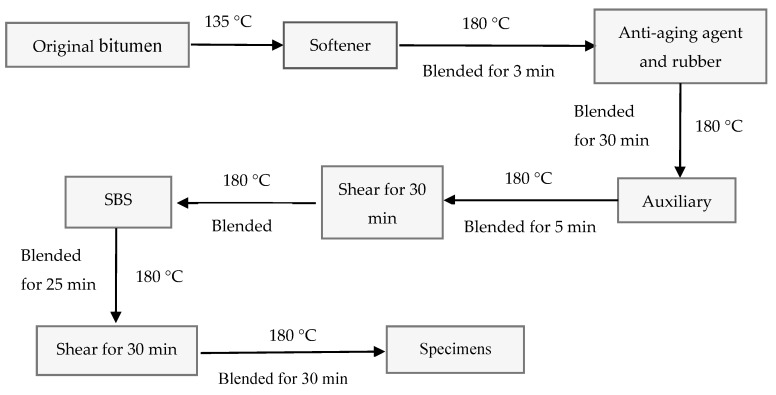
Preparation chart of SBS/CRP-modified bitumen.

The preparation process of SBS/RP-modified bitumen is shown in Figure 1. All specimens were prepared at a temperature of 180 °C. The softeners, anti-aging agents, rubber powder, auxiliaries, and SBS were added into the original bitumen in turn; then, they were sheared using a shearing machine with 5000 rpm and the shearing time was observed during the preparation.

### 2.3. Preparation of Aged Bitumen

The aged bitumen specimens were obtained using a thin film oven test (TFOT), aging at 163 °C for 5 h, ASTM D 1754 and pressurized aging vessel (PAV), aging at 100 °C for 20 h under air of 2.1 MPa, ASTM D 6521, respectively. The TFOT was employed to simulate the short-term aging including the storage, transport, mixing, and paving process of bitumen in pavement construction; the PAV was used to simulate the long-term aging of the bitumen in the service period.

### 2.4. Physical Properties Test

The physical properties of the bitumen samples, including the softening point, penetration at 25 °C, ductility at 15 °C, and viscosity at 135 °C, were tested in accordance with the standards of ASTM D 36, ASTM D 5, ASTM D 113 and ASTM D 6925, respectively. Each specimen was tested three times and the average value was adopted as the testing result.

### 2.5. Dynamic Rheological Property Test

The dynamic rheological properties of the bitumen were measured using a dynamic shear rheometer. The temperature sweeping test of the bitumen was performed under strain-controlled mode at a constant frequency of 10 rad/s. The temperature range was from −20 to 80 °C with a temperature increment of 2 °C per minute. The plate used was 8 mm in diameter and the gap between the parallel plates was 2 mm for each sample below 20 °C. When the temperature was above 20 °C, the plate used was 25 mm in diameter and the gap between the parallel plates was 1 mm.

### 2.6. Performance Test at Low Temperature

The low-temperature performance of original bitumen, SBS-modified bitumen and SBS/CRP-modified bitumen before and after aging was evaluated using the Bending Beam Rheometer (BBR) test. The test temperature was −12 °C, and the loading time and unloading time were 240 s and 10 s, respectively. The test was carried out 3 times and the average value was calculated and used as the testing result. The creep load was used to simulate the accumulated stress step by step in the performance test at low temperature. The time-deformation curve, creep rate m, and stiffness modulus at 60 s were outputted. The stiffness modulus and creep rate of bitumen at 8, 15, 30, 60, 120, and 240 s were calculated. The stiffness modulus (S) indicates the resistance to load for bitumen; and the creep rate (m) is a comprehensive reflection of the curve shape and relaxation ability of bitumen. The stiffness modulus (S) and creep rate (m) can be calculated using Equations (1) and (2), respectively.

(1) $$S(t)=\frac{Pl^{3}}{4bh^{3}v(t)}\;$$

(2) $$m=\frac{d\log S(t)}{d\log(t)}\;$$

where, v(t) is the deformation in the middle of the beam, mm; b is the width of the beam, 12.70 mm ± 0.05 mm; P is the constant load of the beam, 980 mN ± 50 mN; h is the height of the beam, 6.35 mm ± 0.0 5 mm; and l is the span of the beam, 102 mm.

In order to further characterize the rheological properties of the modified bitumen at low temperature, Equation (3) can be used to calculate the relationship between creep compliance and time according to the original data of loading time and deformation of PAV aged bitumen, which is the reciprocal equation of Equation (1). Based on the Burgers model, the viscoelastic parameters can be obtained using Equations (4) and (5) and the comprehensive compliance parameters are used to evaluate the low-temperature relaxation characteristics of bitumen.

(3) $$\;S(t)=\frac{Pl^{3}}{4bh^{3}v(t)}=\frac{1}{D(t)}\;$$

(4) J(t)=1E1+1E2(1−e−tE2η2)+tη1 

The Burgers creep equation is expressed as:

(5) $$J(t)=J_{E}+J_{De}+J_{V}\;$$

where $J_{E}=\frac{1}{E_{1}}$, JDe=1E2(1−e−tE2η2), $J_{V}=\frac{t}{\eta_{1}}$; comprehensive compliance parameter $J=J_{V}(1-\frac{J_{E}+J_{De}}{J_{E}+J_{De}+J_{V}})$ can be used to characterize the low-temperature performance of bitumen materials.

### 2.7. Infrared Spectrum Test

Original bitumen, SBS-modified bitumen and SBS/CRP-modified bitumen after short-term TFOT aging and the PAV pressure aging were tested using infrared spectroscopy so as to further elucidate the relationship between the macroscopic properties and microstructure of bitumen. The wavelength range of infrared spectroscopy was 400–4000 cm^−1^ and the test temperature was 20 °C.

## 3. Results and Discussion

### 3.1. Physical Properties of SBS/CRP-Modified Bitumen

Table 3 shows the properties of three kinds of bitumen after different aging processes. It shows that the addition of modifiers can greatly affect the softening point, viscosity, and penetration of original bitumen. The softening point of SBS-modified bitumen and SBS/CRP-modified bitumen increases by 58.2% and 75.8%, respectively. The penetration decreases by 19.4% and 23.1%, respectively. The rotational viscosity at 175 °C is 6.8 times and 16.9 times of that of original bitumen, respectively. The softening point of original bitumen increases obviously after modification and the penetration decreases slightly, which is mainly due to the formation of the network structure [26] in the original bitumen due to the addition of modifiers and the enhancement of the interaction between bitumen molecules. The viscosity of the modified bitumen increases greatly because the modifier absorbs the lightweight components of the original bitumen and forms the network structure [26], which increases the resistance of the rotary viscometer. In particular, the friction resistance of the rotary viscometer is further increased with the addition of rubber particles. The viscosity of the SBS/CRP-modified bitumen increases greatly with the temperature and the viscosity is greatly reduced.

After short-term aging, the softening point of original bitumen increases by 12.6%; the penetration decreases by 46.2%; and the rotational viscosity at 175 °C increase by 2 times. The softening point of SBS-modified bitumen and SBS/CRP-modified bitumen decreases by 12.4% and 9.5%; the penetration decreases by 65.6% and 37.4%, respectively; and the viscosity is 1.2 times and 1.9 times that of the original bitumen. In contrast to the original bitumen, the softening point of the modified bitumen increases; the penetration decreases; the viscosity increases; and the bitumen gradually becomes hard and brittle. The carbon-carbon double bond of the modified bitumen is broken. The network structure is damaged and the hard segment of styrene is dispersed in the modified bitumen. Therefore, the softening point decreases; the penetration decreases; and the viscosity increases.

After the long-term aging process, the ductility of three kinds of bitumen decreases obviously and the viscosity increases. The softening point of original bitumen and SBS-modified bitumen increases slightly, but the softening point of SBS/CRP-modified bitumen does not decrease significantly. This shows that the flexibility and structure of the SBS/CRP-modified bitumen are still in a good condition after long-term aging and that its anti-aging ability is still strong.

### 3.2. Dynamic Rheological Properties of SBS/CRP-Modified Bitumen

**Figure 2:**
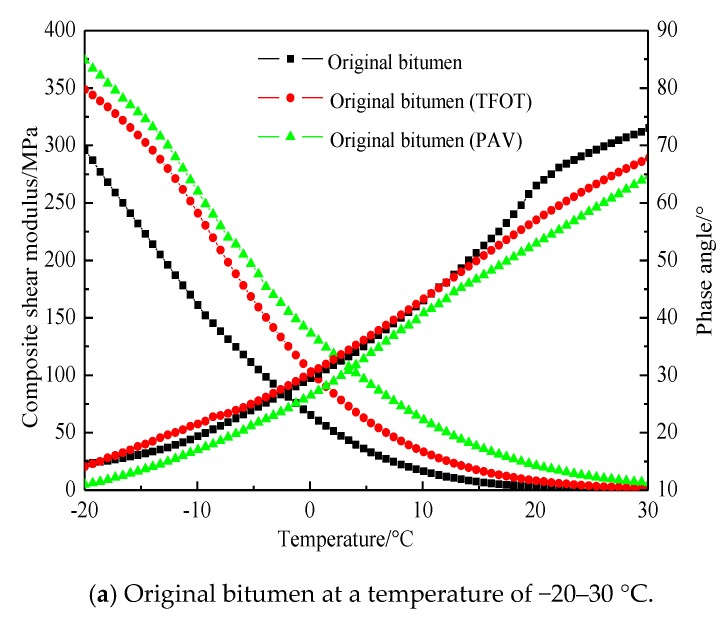

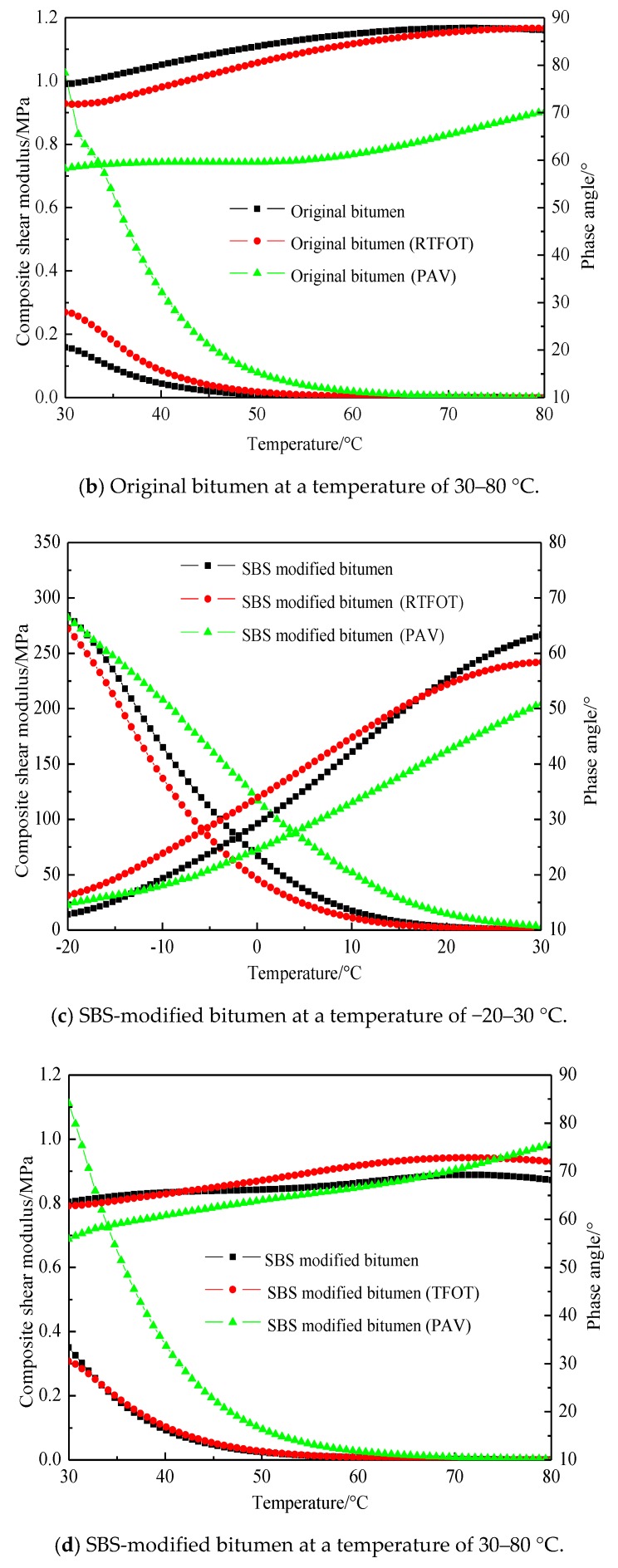

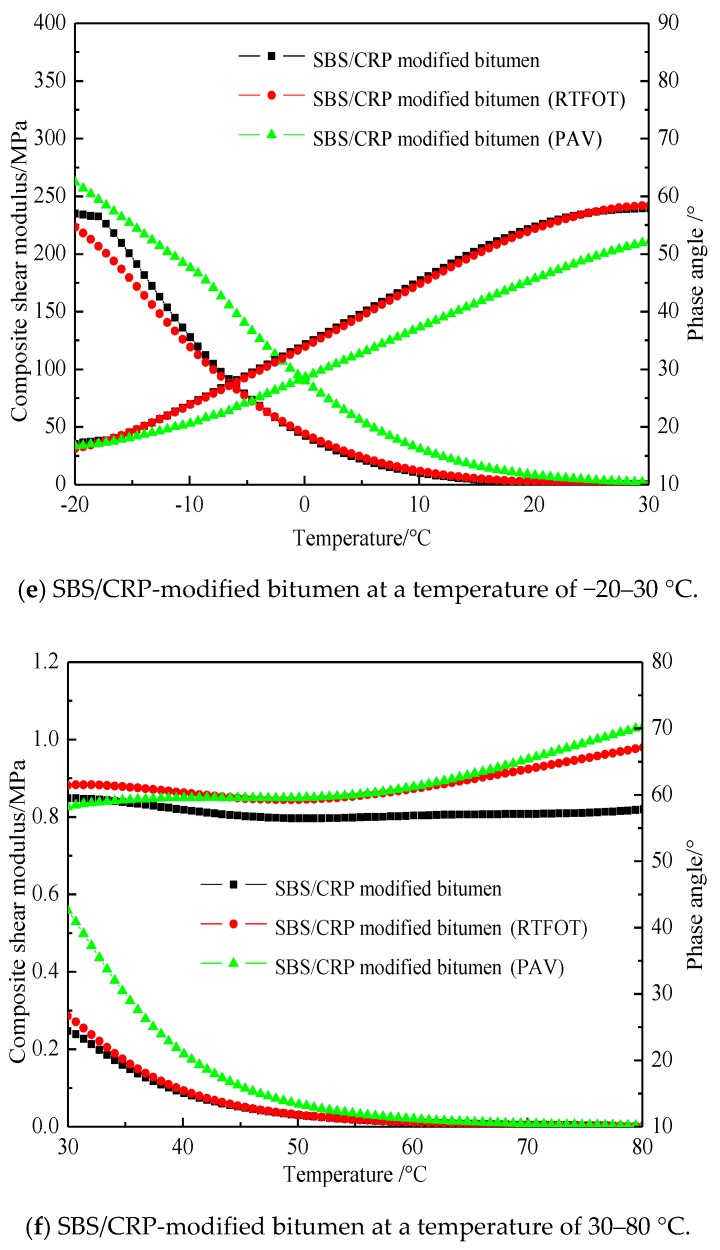
Dynamic rheological properties of bitumen at different temperatures.

Figure 2 shows the composite shear modulus and phase angle of three kinds of bitumen before and after aging. With the increase in temperature, the composite shear modulus of bitumen decreases and the phase angle increases, mainly due to the softening of bitumen and the decrease in viscosity. As shown in Figure 2a,b, the composite shear modulus of bitumen increases and the phase angle decreases after the aging and long-term aging of original bitumen. The phase angles of bitumen before and after aging at the same temperature change slightly during −20–30 °C. That is, the proportion of the bitumen elastic component is larger and it tends to be elastic-plastic at the middle and low temperatures. The variation range of the composite shear modulus before and after aging at −20 and 30 °C is 299–378 MPa and 0.4–6.3 MPa, respectively. After short-term aging, the phase angle of original bitumen at 30–80 °C is smaller than that of the original bitumen, which is 70° and 87°, respectively, but the phase angle of original bitumen is large. The proportion of the viscous part is high in this temperature range. When the temperature is low, the viscosity of three kinds of bitumen after aging is very high, which is close to being elastic-plastic. Moreover, the stress hysteresis becomes weaker after loading. Therefore, the change in phase angle before and after aging is small. When the temperature is relatively high, the temperature sensitivity of the original bitumen is poor. The longer the aging time, the more volatile the light components between the bitumen molecules are, the more severe the heavy ones are, the greater the viscosity, and the weaker the stress hysteresis, which shows that the short-term aging phase angle decreases less and the long-term aging decreases more.

As shown in Figure 2c,d, the change trend of SBS-modified bitumen after short-term aging is different from that of original bitumen. After short-term aging, the composite shear modulus of bitumen decreases and the phase angle increases slightly for SBS-modified bitumen, which is more obvious at −20–18 °C. The curve of 18–80 °C almost coincides with the original curve and the change in the composite shear modulus and phase angle is not obvious, which indicates that the contribution of the composite shear modulus to the storage modulus becomes smaller and smaller; the elasticity of modified bitumen is lower and the viscosity is enhanced. The main reason for this is that the unsaturated double bond of the SBS modifier is broken and partly degraded under the action of thermal oxygen at 163 °C, which destroys its original structure and the elasticity of the modifier. The increase in the phase angle is not obvious after short-term aging. This is mainly due to the fact that the further degradation of the modifier can lead to an increase in the phase angle, while the hardening degree of original bitumen can lead to a decrease in the phase angle. With long-term aging, the elasticity reduction caused by the degradation of the modifier is reduced, and the hardening of the original bitumen is dominant. After long-term aging, the phase angle decreases and the composite shear modulus of bitumen increases.

As shown in Figure 2e,f, the change trend of SBS/CRP-modified bitumen is the same as that of SBS. Short-term aging has little effect on the composite shear modulus or phase angle of SBS/CRP-modified bitumen. The curves of unaged and short-term aged bitumen almost coincide at 40 °C. In contrast to SBS-modified bitumen at the same temperature, SBS/CRP-modified bitumen possesses a smaller composite shear modulus, which indicates that SBS/CRP-modified bitumen has lower strength and greater flexibility at a low temperature. The composite shear modulus and phase change with the change in temperature and the temperature sensitivity are good. The main reason for this is that a three-dimensional network structure is formed in the compound modification system of SBS and rubber powder [27]. When the strain of SBS-modified bitumen reaches the limit state, the fracture stress can quickly concentrate on the surface of crumb rubber powder particles. Then, the crumb rubber powder particles absorb and consume a large amount of energy, which prevents the crack from forming and expanding. At the same time, crumb rubber powder particles play a reinforcing role in bitumen, meaning that the elastic recovery ability of the two composite modifications can be significantly improved, which shows a strong low-temperature deformation capacity.

### 3.3. Performance of SBS/CRP-Modified Bitumen at Low Temperature

#### 3.3.1. Effects of Aging on Stiffness Modulus and Creep Rate

**Figure 3:**
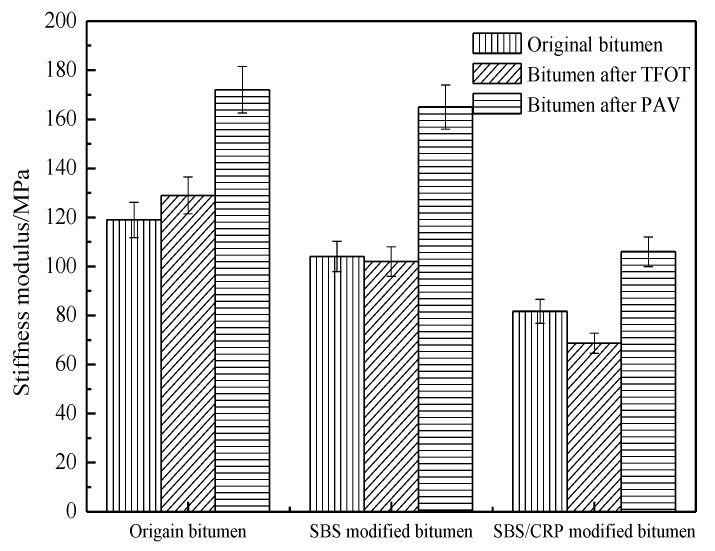
The effects of aging on the stiffness modulus of bitumen.

**Figure 4:**
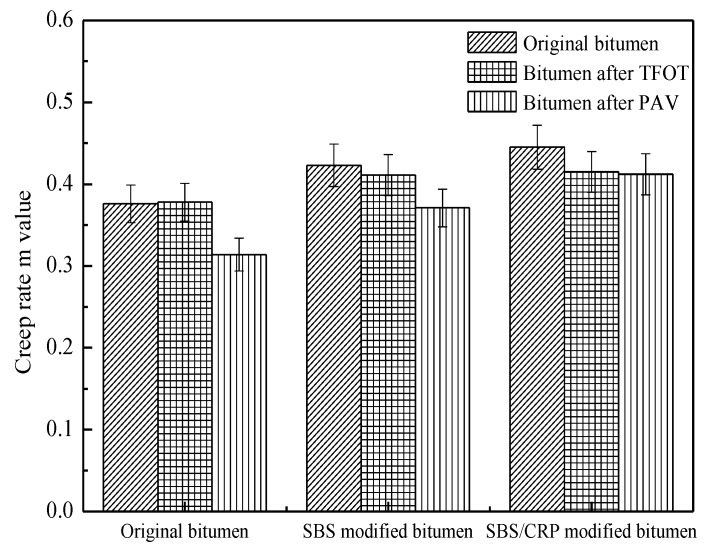
The effects of aging on the creep rate of bitumen.

Figure 3 and Figure 4 show the change in the stiffness modulus and creep rate of bitumen. As shown in Figure 3, in contrast to the original bitumen after short-term aging, the stiffness modulus of original bitumen increases, while those of SBS-modified bitumen and SBS/CRP-modified bitumen decrease, which is consistent with the results of the composite shear modulus. The stiffness modulus of the three bitumen increases significantly after long-term aging. The main reason for this is that the original bitumen tends to be in densification during thermal-oxygen aging and pressure aging and the original bitumen shows brittle and rigid characteristics, which result in poor low-temperature relaxation ability of original bitumen. As shown in Figure 4, after short-term aging, the m value of original bitumen increases slightly, while SBS-modified bitumen and SBS/CRP-modified bitumen decrease, which is inconsistent with the result of stiffness modulus. Moreover, the creep rate of all kinds of bitumen decreases after long-term aging. However, there are some limitations in evaluating the low-temperature performance of bitumen by S and m values at a certain test temperature. The creep properties of bitumen based on the Burgers model can be used to evaluate the low-temperature performance of bitumen.

#### 3.3.2. Stiffness Modulus Curve of Bitumen

**Figure 5:**
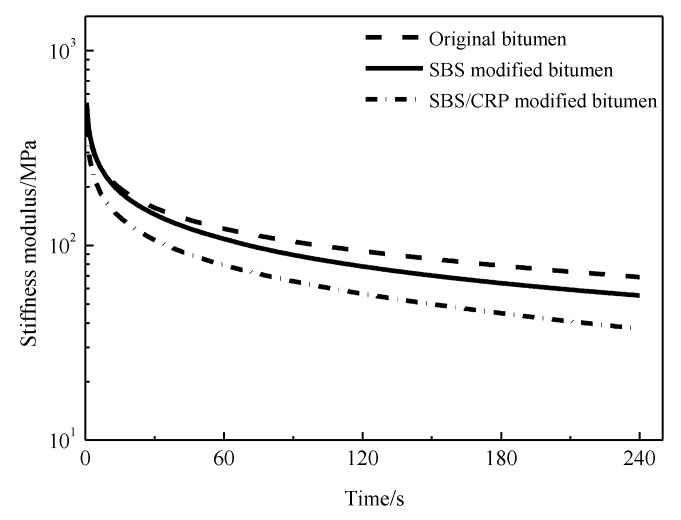
Creep stiffness modulus versus time curve of bitumen.

The stiffness modulus curves of three kinds of bitumen after PAV are shown in Figure 5, which can be calculated using the original data of performance at low temperature test. As shown in Figure 5, the logarithm of the creep stiffness modulus decreases gradually with a time delay in the process of constant force loading, and the higher the slope of creep stiffness is, the stronger the creep ability is. Three kinds of creep stiffness modulus slope in the following order: original bitumen < SBS-modified bitumen < SBS/CRP-modified bitumen. At the same temperature, the stiffness modulus of SBS-modified bitumen is close to that of original bitumen; however, the difference between SBS-modified bitumen and SBS/CRP-modified bitumen is higher than that of original bitumen. And the difference between SBS-modified bitumen and SBS/CRP-modified bitumen is more obvious with the decrease in temperature, which further indicates that SBS/CRP-modified bitumen has a stronger low-temperature relaxation ability and better low-temperature crack resistance.

#### 3.3.3. Low-Temperature Creep Properties of Bitumen Based on the Burgers Model

Table 4 shows the Burgers viscoelastic parameters of different kinds of bitumen. At the same temperature, as the aging degree deepens, the stiffness modulus S of bitumen increases and the creep rate m decreases. Based on the data of Table 4, the comprehensive compliance parameters of different kinds of bitumen are obtained using Equation (5), as shown in Table 5. At a low temperature, the proportion of elastic deformation is bigger and the proportion of viscous deformation is smaller. If the proportion of viscous flow is high, the compressive tensile stress of bitumen pavement can be relaxed by means of flow, and the low-temperature shrinkage cracking of bitumen pavement is reduced. As shown in Table 5, the J value of SBS-modified bitumen and original bitumen is 1.04 times and 1.55 times, respectively.

### 3.4. Infrared Spectrum Analyses of SBS/CRP-Modified Bitumen

**Figure 6:**
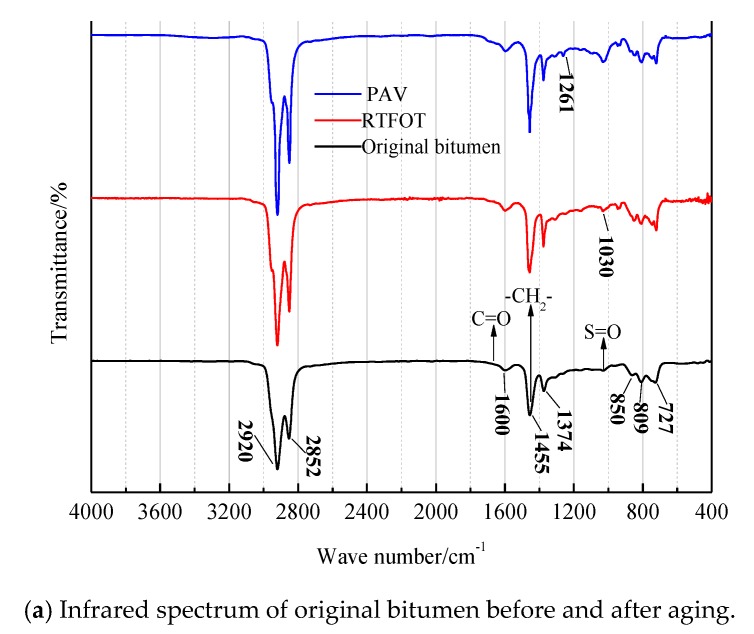

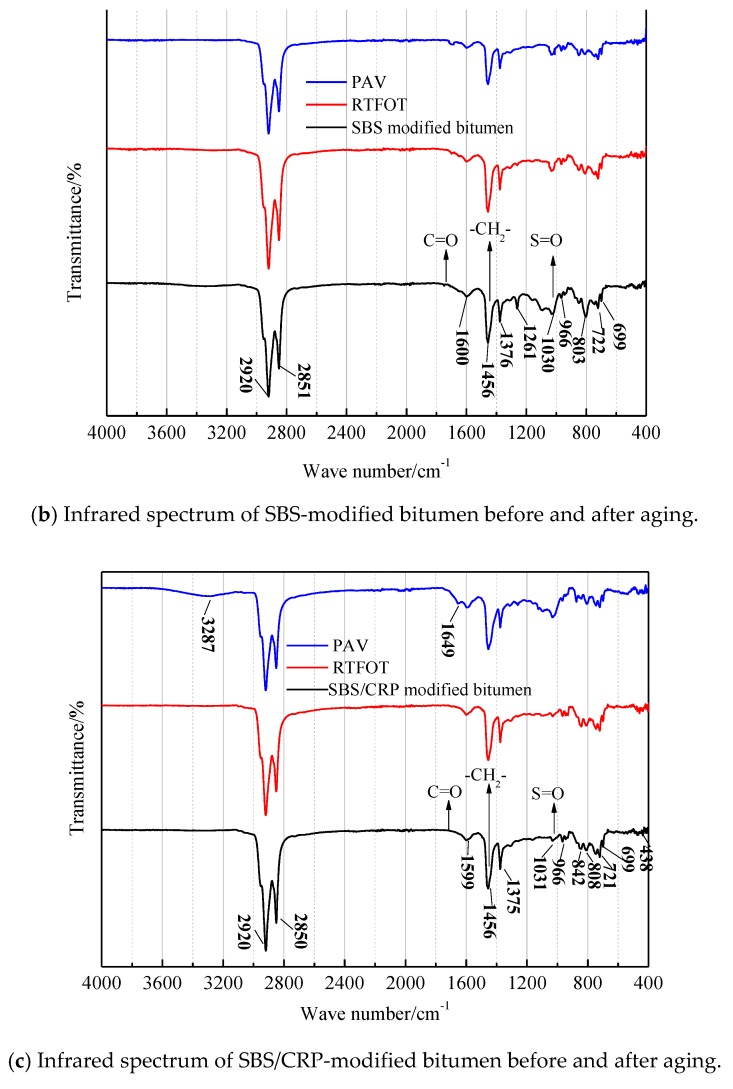
Infrared spectra of three kinds of bitumen before and after aging.

Figure 6 shows the infrared spectra of different kinds of bitumen before and after aging. There are eight obvious characteristic absorption peaks in the infrared spectra of three kinds of bitumen before and after aging. The corresponding wave numbers are 2920 cm^−1^, 2852 cm^−1^, 1600 cm^−1^, 1455 cm^−1^, 1374 cm^−1^, 850 cm^−1^, 809 cm^−1^, and 727 cm^−1^, respectively. The strong absorption peaks of 2920 cm^−1^ and 2852 cm^−1^ are the reverse stretching peaks and the methylene symmetrical stretching peaks of aliphatic methylene C–H with acromion. The wave number 1600 cm^−1^ is the stretching vibration of the aromatic C=C skeleton. The wave numbers 1455 cm^−1^ and 1374 cm^−1^ are the bending vibration absorption peaks of methylene (–CH_2_^−^) and methyl (–CH_3_), respectively, which prove the existence of long carbon chains. In the wave numbers 2920 cm^−1^ and 1455 cm^−1^ in three kinds of bitumen, C–H is the main chain. The wave number 650–900 cm^−1^ is called the benzene ring substituted region. The wave numbers 809 cm^−1^ and 727 cm^−1^ are the absorption peaks of C–H surface bending vibration on the aromatic benzene ring. As shown in Figure 6a, the absorption peaks of sulfoxide S=O at 1030 cm^−1^ and carbonyl at 1600 cm^−1^ are enhanced. The absorption magnitudes of 2920 cm^−1^, 2852 cm^−1^, and 1455 cm^−1^ are also enhanced. The transmittance of the sulfoxide group after PAV aging is stronger than that after TFOT aging. In addition, the characteristic peaks representing methylene 1261 cm^−1^ also appear after the PAV aging process.

Therefore, the aging process of bitumen is characterized by the increase in the methyl, methylene, sulfoxide, and carbonyl peaks, which indicates that the long chain of bitumen is broken; the short chain is dehydrogenated and condensed; and the active functional groups in the bitumen molecules combine with oxygen molecules in the air to form polar macromolecules such as sulfoxide and carbonyl. The sulfoxide group S=O increases obviously after long-term aging, and the sulfoxide group has poor thermal stability. Sulfur is added to the bitumen directly when the bitumen reacts with sulfur at 140 °C and it can decompose when the temperature rises. When the temperature rises to 180 °C, hydrogen sulfide and other substances are produced, which can join with sulfur to form macromolecules. When the temperature continues to rise, the dehydrogenation reaction occurs and the S=O is generated.

As shown in Figure 6b, in contrast to original bitumen, the absorption peak of 966 cm^−1^ of SBS-modified bitumen increases, which is the absorption peak corresponding to the C=C bond of butadiene in SBS-modified bitumen; and the absorption peak of 966 cm^−1^ goes down after aging. The results show that the unsaturated carbon-carbon double bond of the SBS modifier breaks after short-term aging and long-term aging; and the network structure of SBS is destroyed. With the increase in temperature, the interaction between the bitumen molecules becomes weaker; the softening point decreases, the composite shear modulus and stiffness modulus decrease slightly; and the hard segment of styrene is dispersed in the modified bitumen. In SBS-modified bitumen, the penetration decreases and the carbon-carbon double bonds break after long-term aging, tending to the variation of original bitumen. The composite shear modulus and stiffness modulus increase gradually, and the relaxation ability of SBS-modified bitumen decreases.

However, the characteristic peaks of SBS/CRP-modified bitumen do not change significantly compared with the SBS-modified bitumen, as shown in Figure 6c. After long-term aging, the absorption peaks of 3287 cm^−1^ and 1649 cm^−1^ appear. The wave number 3287 cm^−1^ can be generated by hydroxyl in air and 1649 cm^−1^ is generated by oxidized carbonyl.

## 4. Conclusions and Recommendations

In this study, SBS/CRP-modified bitumen was prepared. Temperature sensitivity performance, such as the high-temperature performance and low-temperature performance of original bitumen, SBS-modified bitumen and SBS/CRP-modified bitumen under different aging processes were evaluated and analyzed. Additionally, the relationship between macroscopic properties and microstructures of SBS/CRP-modified bitumen was discussed. Then, the following conclusions were drawn:

(1) The addition of modifiers can increase the softening point and viscosity and decrease the penetration of original bitumen. The ductility of three kinds of long-term aged bitumen decreases obviously and the viscosity increases. The softening point of aged original bitumen and aged SBS-modified bitumen increases slightly, but the softening point of SBS/CRP-modified bitumen does not decrease significantly. This shows that SBS/CRP-modified bitumen possesses a strong anti-aging ability in that its flexibility and structure remain in a good condition after long-term aging.
(2) In low-temperature surroundings, the stiffness modulus of SBS/CRP-modified bitumen is the lowest and the J value is the highest among three kinds of bitumen. That is to say, SBS/CRP-modified bitumen has excellent low-temperature relaxation ability and low-temperature crack resistance.
(3) In contrast to original bitumen and SBS-modified bitumen, SBS/CRP-modified bitumen possesses a stronger anti-aging ability. The composite shear modulus and phase angle fluctuation of SBS/CRP-modified bitumen with temperature change slightly, and the temperature sensitivity performance of SBS/CRP-modified bitumen decreases.
(4) The aging process of original bitumen is characterized by an increase in methyl, methylene, sulfoxide, and carbonyl peaks, and that of SBS-modified bitumen is close to that of original bitumen. After long-term aging, the wave numbers 3287 cm^−1^ and 1649 cm^−1^, the characteristic peaks of SBS/CRP-modified bitumen, increase, where 3287 cm^−1^ can be the hydroxyl group formed in the air and 1649 cm^−1^ is the carbonyl group formed by peroxidation. The physical blending effect is dominant and there is no evident chemical reaction between bitumen and crumb rubber powder.
(5) In contrast to original bitumen and SBS-modified bitumen, SBS/CRP-modified bitumen can be recommended for use in plateau areas. Of course, it is suggested that the low-temperature properties of SBS/CRP-modified bitumen under different temperatures be studied in future research.

## Figures and Tables

**Table 1:** Properties of original bitumen.

Properties		Unit	Test Results	Specification	Test Method
Penetration (25 °C, 100 g, 5 s)		0.1 mm	94.6	80–100	T0604
Ductility (15 °C, 5 cm/min)		cm	>100	≮100	T0605
Softening point (Ring-and-ball method)		°C	45.8	≮44	T0606
After TFOT	Mass loss	%	+0.4	±0.8	T0610
	Residual penetration (25 °C)	%	57.8	≮57	T0604
	Residual ductility (10 °C)	cm	12	≮8	T0605

**Table 2:** Properties of crumb rubber powder.

Properties		Unit	Test Results
Physical properties	Density	g/cm^3^	1.15
	Moisture	%	0.46
	Metal content	%	0.005
	Fiber content	%	0.52
Chemical properties	Minerals	%	6.1
	Acetone extract	%	7.1
	Carbon black content	%	30
	Rubber hydrocarbon	%	50.1

**Table 3:** Properties of bitumen with different aging processes.

Bitumen Type		Ductility/cm		Softening Point/°C	Penetration/0.1 mm	Rotational Viscosity at 135 °C/mPa·s	Rotational Viscosity at 175 °C/mPa·s
		5 °C	10 °C				
Bitumenbefore aging	Original	-	54.6	46.7	92.1	353	56
	SBS	34.6		73.9	74.2	1400	382
	SBS/CRP	30.5		82.1	70.8	4300	945
Bitumenafter TFOT	Original	-	8.0	52.6	49.5	507	101
	SBS	23.0		64.7	44.8	2440	460
	SBS/CRP	15.1		75.2	51.5	6110	1820
Bitumenafter PAV	Original	-	4.2	60.7	29.8	1010	207
	SBS	1.5		65.1	21.4	3567	670
	SBS/CRP	6.0		73.4	35.0	6700	1900

**Table 4:** Burgers viscoelastic parameters of different kinds of bitumen.

	Bitumen Type	Original Bitumen	SBS-Modified Bitumen	SBS/CRP-Modified Bitumen
Parameters				
E_1_/MPa		9000.0	8000.0	7000.0
E_2_/MPa		617.3	342.6	266.1
η_1_/MPa·s		40,971.9	52,584.8	23,164.1
η_2_/MPa·s		7282.8	6781.8	4605.7

Note: The values of E_1_ were obtained using the empirical method: see Reference [28].

**Table 5:** Comprehensive creep compliance parameters of different kinds of bitumen.

	Bitumen Type	Original Bitumen	SBS-Modified Bitumen	SBS/CRP-Modified Bitumen
Index/GPa				
J_E_/(1/MPa)		1.11 E-04	1.25 E-04	1.43 E-04
J_De_/(1/MPa)		1.62 E-03	2.92 E-03	3.76 E-03
J_V_/(1/MPa)		3.30 E-02	3.54 E-02	5.21 E-02
J/(1/MPa)		3.13 E-02	3.26 E-02	4.85 E-02
